# Obesity Exacerbates the Cytokine Storm Elicited by* Francisella tularensis* Infection of Females and Is Associated with Increased Mortality

**DOI:** 10.1155/2018/3412732

**Published:** 2018-06-26

**Authors:** Mireya G. Ramos Muniz, Matthew Palfreeman, Nicole Setzu, Michelle A. Sanchez, Pamela Saenz Portillo, Kristine M. Garza, Kristin L. Gosselink, Charles T. Spencer

**Affiliations:** Department of Biological Sciences and Border Biomedical Research Center, University of Texas at El Paso, El Paso, TX, USA

## Abstract

Infection with* Francisella tularensis*, the causative agent of the human disease tularemia, results in the overproduction of inflammatory cytokines, termed the cytokine storm. Excess metabolic byproducts of obesity accumulate in obese individuals and activate the same inflammatory signaling pathways as* F. tularensis *infection. In addition, elevated levels of leptin in obese individuals also increase inflammation. Since leptin is produced by adipocytes, we hypothesized that increased fat of obese females may make them more susceptible to* F. tularensis* infection compared with lean individuals. Lean and obese female mice were infected with* F. tularensis* and the immunopathology and susceptibility monitored. Plasma and tissue cytokines were analyzed by multiplex ELISA and real-time RT-PCR, respectively. Obese mice were more sensitive to infection, developing a more intense cytokine storm, which was associated with increased death of obese mice compared with lean mice. This enhanced inflammatory response correlated with* in vitro* bacteria-infected macrophage cultures where addition of leptin led to increased production of inflammatory cytokines. We conclude that increased basal leptin expression in obese individuals causes a persistent low-level inflammatory response making them more susceptible to* F. tularensis* infection and heightening the generation of the immunopathological cytokine storm.

## 1. Introduction

Infection with the zoonotic pathogen* Francisella tularensis*, the causative agent of human tularemia, elicits a profound overproduction of inflammatory cytokines, culminating in a cytokine storm. Following macrophage uptake of the bacterium,* F. tularensis* escapes into the cytosol where it initiates inflammation [1, 2]. During lethal infection, excessive levels of proinflammatory cytokines, e.g., IFN-*γ*, TNF-*α*, and IL-6, are observed in the plasma indicative of a systemic sepsis-like response [3, 4]. This results in excessive immunopathology, including loosening of endothelial tight junctions, edema, hypovolemia, fever, and bradycardia. While low levels of inflammation are necessary to activate the immune response and clear pathogen infection, including* F. tularensis*, excessive inflammation causes this lethal immunopathology.

Excess caloric intake leads to the swelling of adipocytes and the activation of local adipose tissue leukocytes [5–9]. This activation results in increased production of proinflammatory cytokines and adipokines by macrophages and T cells in an attempt to control and remove excess fat and fat-swollen cells [10, 11]. Activation of adipose tissue macrophages results in IL-1*β* secretion, which in turn triggers production of inflammatory cytokines and adipokines [12–17]. Persistent hypertrophic stress on adipocytes causes continuous production of inflammatory and anti-inflammatory cytokines and adipokines [18–20] and a chronic low-level inflammation in obese individuals [21–24]. Furthermore, extra stores of adipocytes in females are thought to contribute to their increased basal inflammatory response. However, this inflammation is not limited to local adipose tissue as leptin, released into the blood plasma, can activate other immune cells [25]. Leptin receptor is found on T and B lymphocytes, as well as monocytes and macrophages, and stimulates proinflammatory functions of these distant immune cells [12, 13, 26–30].

While obesity increases the risk of several infectious diseases, limited or controversial data exists on its role in an individual's predisposition for bacteremia and sepsis or the severity of the cytokine storm [31, 32]. Since both obesity and* F. tularensis* activate the same inflammatory signaling pathways, we hypothesized an additive activation of inflammation elicited by* F. tularensis* infection of obese individuals. This increased inflammation would then result in an increased cytokine storm and, therefore, increased susceptibility to* F. tularensis*-mediated disease.

## 2. Materials and Methods

### 2.1. Ethics Statement

All animal procedures were carried out in accordance with the Guide for the Care and Use of Laboratory Animals and approved by the Institutional Animal Care and Use Committee under protocol A-201208-1.

### 2.2. Animals

Age-matched female C57BL/6J mice were purchased from Jackson Laboratories (Bar Harbor, ME) and maintained on a 12h light/dark cycle with* ad libitum* access to food and water. Diet-induced obesity was caused by providing chow containing 60% kcal from fat for nine weeks (Envigo, Houston, TX). Female mice were selected for this study because of past experience in infectious disease and obesity studies. In a forthcoming publication, we identified a profound difference in the susceptibility of male and female mice to* F. tularensis* infection independent of obesity and obesity-related signaling. In addition, male and female mice deposit excess fat at different rates as well as have profound differences in adipokine production [33]. Furthermore, females have increased severity and mortality from sepsis compared to males [34, 35]. Therefore, in order to focus on the effects of obesity alone, female mice were chosen for this study as the sex more sensitive to sepsis-like disease.

### 2.3. Bacteria

*Francisella tularensis* subspp.* holarctica* LVS (Denise Monack, Stanford University) was grown on chocolate agar plates for 24-48 hours. Plates were scraped aseptically and the organisms harvested into sterile PBS with 20% glycerol and stored at -80°C. Concentrations of thawed aliquots were subsequently determined by serial dilution and used for all aliquots.

### 2.4. Animal Infection

For injection, bacterial stocks were diluted to the indicated concentration in sterile PBS. Mice anesthetized with 3-5% isoflurane inhalation were injected intradermally in the flank above the hind quarters with 50ul of bacteria diluted in PBS. Animals were monitored every 12 hours postinfection for the first 48 hours and every 8 hours thereafter. All animals were weighed before inoculation and every morning thereafter. An animal was considered terminal and humanely euthanized per AVMA standards when it had lost 20% of its baseline weight. In addition, animals were checked for clinical symptoms of disease and considered terminal when lethargic and immobile with prodding.

### 2.5. Blood Draw

Blood was drawn on days 0, 3, and 5 after infection from the retroorbital capillary sinus using heparinized capillary tubes and at the time of euthanasia (T) by cardiac puncture. As each animal was bled every other day, alternating eyes were used to prevent irritation and ocular damage. Whole blood was fractionated and plasma frozen until completion of the experiment for subsequent analyses.

### 2.6. Plasma Cytokine Analysis

Concentrations of cytokines and chemokines in the plasma of infected animals were determined by multiplex ELISA (MilliPlex, Millipore Sigma, St. Louis, MO) and analyzed on a Luminex MagPix (Austin, TX) following manufacturer's protocols. Analysis was completed on individual animals at each time point and analyzed by multiparametric t-test.

### 2.7. Real-Time RT-PCR

At the time of euthanasia, spleen, liver, and lung tissues were harvested, mechanically dissociated, and submerged in RNAlater. Subsequently, RNA was extracted using an RNEasy kit (Qiagen, Germantown, MD) and analyzed by real-time RT-PCR using the CYBRFast 1-step RT-qPCR kit (Tonbo Biosciences, San Diego, CA) and the StepOne Real-Time PCR System (Applied Biosystems, Foster City, CA). ΔCt values were calculated by comparison with GAPDH expression levels and ΔΔCt values by comparison with the average Ct value from uninfected tissue and are reported as fold changes in expression.

### 2.8. In Vitro Inflammatory Assay

Immortalized C57BL/6 bone-marrow derived macrophages (BEI Resources, NIAID) were seeded at 7.5x10^5^ per well in 96-well plates in the presence or absence of 2 ug/ml leptin (Millipore Sigma). Macrophages were chosen due to the tropism of the bacterium for these cells and the ability to culture them* in vitro*. While* F. tularensis* also infects neutrophils* in vivo*, the neutrophil lifespan (12-36 hours) is too short for our* in vitro* culture model which lasts ~60 hrs. In addition, leptin has been shown not to alter the production of inflammatory cytokines from neutrophils following purification [36]. After attachment overnight, macrophages were infected with a multiplicity of infection (MOI)=40 LVS bacteria for 2 hours followed by wash and addition of 20 ug/ml gentamicin containing medium to kill extracellular bacteria and prevent overgrowth of the wells. During all procedures, leptin was continually present in the medium at 2 ug/ml in appropriate wells. 48 hours after bacterial inoculation, the production of cytokines by macrophages in response to infection was determined by MilliPlex.

### 2.9. Statistical Analysis

Group weights were compared by Mann–Whitney U test, while survival curves of those groups were compared using Mantel-Cox test with 10-15 animals per group. Plasma cytokine levels were analyzed either by ANOVA with Tukey's posttest or multiparametric t-test for repeated sampling measures. Levels of* in vitro *inflammation were compared by Mann–Whitney U test. Significance was determined at the p≤0.05 level. Statistical analyses and graphs were generated using GraphPad Prism.

## 3. Results

**Figure 1:**
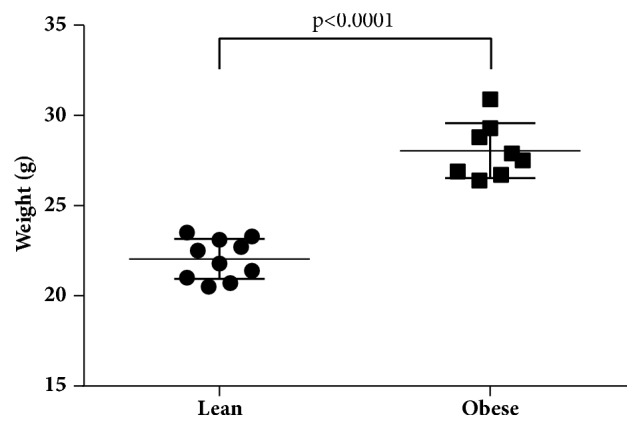
Development of obesity: C57BL/6J mice fed high fat chow for 9 weeks were, on average, 10g heavier (p<0.0001, n=15) than their sibling lean counterparts. Data is representative of three replicate experimental groups.

Following 9 weeks of high fat chow, female mice were, on average, 10g heavier than control mice fed normal fat chow (Figure 1; p<0.0001 by Mann–Whitney U test). Little difference was detectable in the blood levels of 20 different cytokines involved in various immunological pathways prior to infection (Figure S1). Serum levels of IL-6, TNF-*α*, GM-CSF, MIP-3*α*, and IL-22 were slightly elevated, while IL-15 was strongly reduced (Table S1). However, the only statistically significant differences observed were decreases in the circulating levels of sCD40L and IL-21 in obese animals (Figure S2).

**Figure 2:**
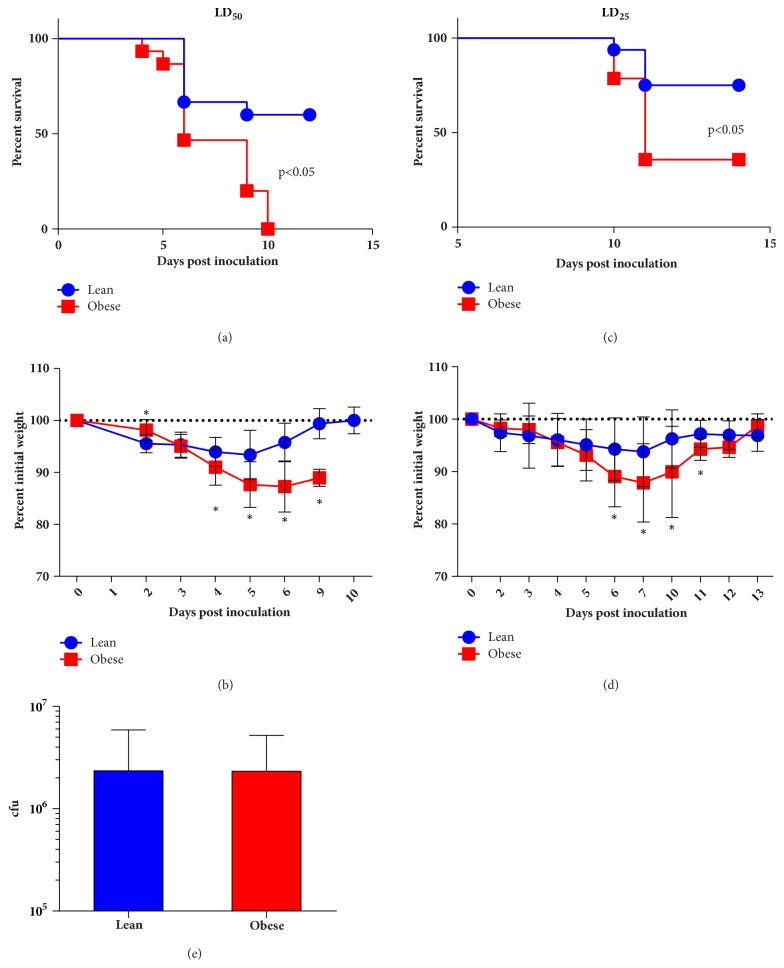
Increased susceptibility of obese animals following* F. tularensis* infection. Lean and obese mice were infected with 8x10^5^ (a, b) or 5x10^5^ (c, d) cfu* F. tularensis* LVS (*∗* (a, c) p<0.05 by Mantel-Cox test; (b, d) p<0.02 by multiple t-test). (e) Bacterial load was indistinguishable in target organs between lean and obese animals.

Lean and obese female mice were inoculated with 8x10^5^ cfu* F. tularensis *LVS (LD_50_ for female C57BL/6) and monitored for the following 14 days. While 50% of lean animals survived the infection, none of the obese animals survived (Figure 2(a); p<0.05 by Mantel-Cox). Daily weight measurements demonstrated that, as a group, obese mice lost 10% more body weight than lean mice (Figure 2(b); p<0.02 by multiple t-test). Decreasing the infectious dose of* F. tularensis *increases survival in wildtype lean mice. Similarly, administration of a 5x10^5^ cfu* F. tularensis* (LD_25_) resulted in a 75% survival for lean mice, while 35% of obese mice survived (Figure 2(c); p<0.05 by Mantel-Cox). Both lean and obese mice became ill at this dose as shown by body weight loss monitoring with obese mice losing 7% more body weight then lean mice (Figure 2(d); p<0.05 by multiple t-test). Increased susceptibility of obese mice was independent of bacterial load, as splenic burden showed no differences in bacterial load (Figure 2(e); ns by Mann-Whitne).

**Figure 3:**
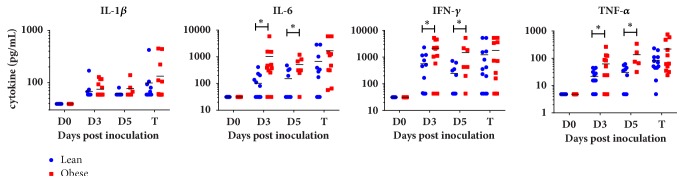
Increased proinflammatory cytokines storm cytokines present in plasma of obese animals. In response to* F. tularensis* initiation, obese mice had significantly higher plasma levels of the inflammatory cytokines compared with lean siblings (*∗*p<0.05 by multiple t test).

**Figure 4:**
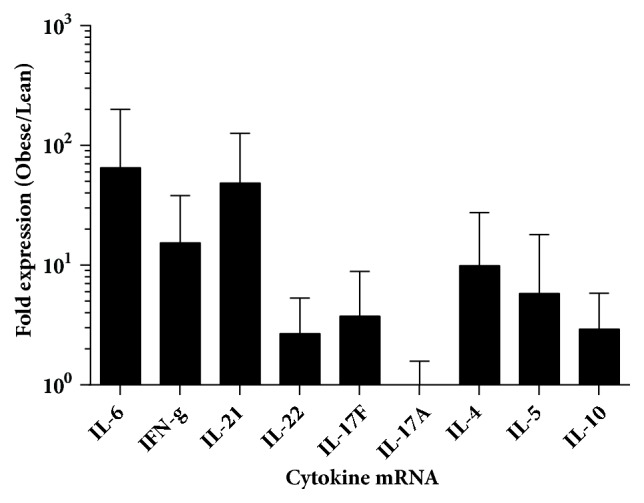
Elevated inflammatory mRNA levels in infected tissues of obese animals. Real-time RT-PCR analysis of mRNA of lean and obese tissue revealed elevated transcript levels of cytokine genes corresponding to cytokine storm cytokines. Data is presented as fold change (2＾-ΔΔCt) in expression in obese animals compared with lean animals (N=15-24).

*F. tularensis *infection results in a septic-like response marked by excessive production of inflammatory cytokines. Therefore, temporal analysis of 19 cytokines and chemokines involved in inflammation and the immune response was performed on individual mice. This demonstrated that obese mice infected with* F. tularensis* had significantly higher plasma levels of IL-6, IFN-*γ*, and TNF-*α* compared with infected lean animals (Figure 3, Table S1; p<0.05 by multiple t-test). This increased proinflammatory response was corroborated by cytokine mRNA transcript expression in infected tissues (Figure 4). Expression of most, but not all, cytokines was upregulated in obese compared with lean mice. In particular, the expression of IL-6, IFN-*γ*, and IL-21 genes was markedly upregulated, with levels 65-, 15-, and 48-fold higher in obese mice compared with lean tissues, respectively.

**Figure 5:**
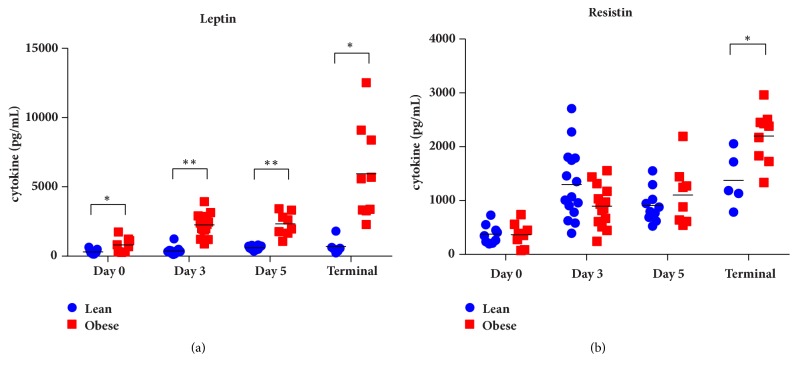
Plasma levels of proinflammatory adipokines. (a) Obese mice had significantly higher plasma levels of the inflammatory adipokine leptin compared with lean siblings (*∗*p<0.05, *∗∗*p<0.001 by multiple t-test). (b) A trend was observed for higher plasma levels of resistin in obese animals compared with lean animals which became significant at the time of death (*∗*p<0.05).

Adipocyte hypertrophy triggers the production of the adipokines leptin, resistin, and adiponectin. Indeed, obese mice infected with* F. tularensis *had elevated levels of the inflammatory adipokines leptin (p<0.005 by multiple t-test) and a trend for increase production of resistin which became significant at the time of termination (Figure 5, Table S2). In addition, there was a general suppression of the anti-inflammatory adiponectin in infected obese mice compared with lean animals during the peak of the inflammatory response though this did not reach statistical significance (Table S2).

**Figure 6:**
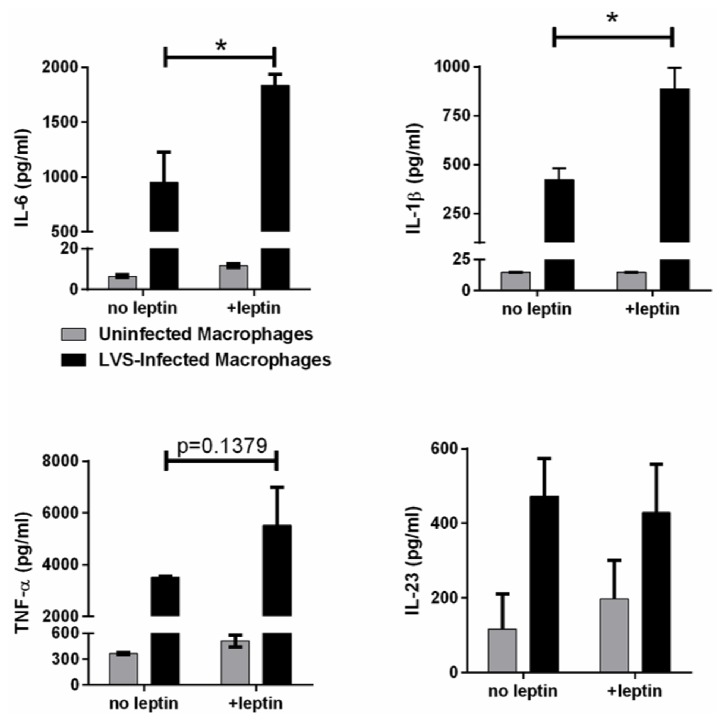
Leptin enhances inflammatory cytokine production by* F. tularensis*-infected macrophages. Macrophages were exposed to leptin prior to, during, and after infection with* F. tularensis *LVS and the production of IL-6 measured as an indicator of inflammation (*∗*p=0.03 by Mann–Whitney t-test).

The leptin receptor is expressed by nearly all immune cells and binding of leptin increases production of Th1 and Th17 responses including IL-6, TNF-*α*, and IFN-*γ*. Therefore, the direct effects of leptin on macrophage inflammation were determined by* in vitro *culture of* F. tularensis*-infected macrophages.* F. tularensis* triggers inflammation in cultured macrophages observed by the production of IL-6, IL-1*β*, TNF-*α*, and IL-23 (Figure 6). Consistent with other studies, addition of leptin to uninfected macrophages at concentrations similar to plasma levels of infected obese mice stimulated a mild increase in the production of these inflammatory cytokines. However,* F. tularensis-*infected macrophages produced significantly higher amounts of IL-6 and IL-*β* compared with infected macrophages in the absence of leptin (Figure 6; p<0.03 by Mann–Whitney t-test). In addition, there was a trend for increased production of TNF-*α* which did not reach statistical significance (p=0.1379 by Mann-Whiteny t-test), while the production of IL-23 was unchanged by the presence of leptin. Together, these data suggest that leptin production from hypertrophic adipocytes in obese mice leads to a heightened inflammation which is exacerbated by the lethal cytokine storm elicited by* F. tularensis *and is associated with increased risk of death following* F. tularensis *infection.

## 4. Discussion

The immune dysfunction caused by obesity has been linked to an increased susceptibility to a number of infection diseases [37]. However, association studies between obesity and sepsis have had mixed results with either no association, positive association, or negative associations being reporting, reviewed in [38].* F. tularensis *infection causes disease through overactivation of the inflammatory response, the cytokine storm, resulting in a sepsis-like disease [3, 4]. Disease symptoms are caused by side effects of the cytokine storm resulting in severe immunopathology.

While the effects of obesity on sepsis remain controversial, leptin's detrimental role in sepsis has been documented in several studies. Results from a multinational European survey of sepsis occurrence in acutely ill patients uncovered that although men present more frequently with sepsis (63%), females were more likely to develop severe sepsis and have higher mortality (OR =1.4) [34]. Furthermore, females have ~2.5 times higher serum levels of leptin compared to males [39]. Since leptin is an inflammatory adipokine, these associations suggest that leptin could be associated with septic symptoms. Indeed, while associative studies demonstrate no link between occurrence of sepsis and leptin, there was a very strong association in females between leptin and severe sepsis, including death following hospitalization (OR=4.18) [39].

Likewise, hyperleptinemia is associated with increased sensitivity to multiple infectious diseases [40]. In addition, the role of leptin in inflammation and inflammatory conditions has been investigated in multiple models [41–43]. However, it remained unknown whether obesity would affect susceptibility to* F. tularensis *infection. Our data demonstrate an increased susceptibility to this infectious disease through enhanced severity of the cytokine storm. While the exact mechanism of disease is unclear for* F. tularensis *infection, it is clearly a septic response [3, 4]. Our data suggest that the mechanism is independent of IL-6-mediated production of CRP as there was no difference in serum CRP levels between lean and obese infected animals despite differing degrees of disease (data not shown).

As reported here, and elsewhere, the cytokine storm elicited by* F. tularensis* is not solely restricted to proinflammatory cytokines but also includes anti-inflammatory and regulatory cytokines and chemokines. The presence of both proinflammatory and anti-inflammatory/regulatory cytokines is a hallmark of the immune dysregulation seen during the cytokine storm. It is currently unknown whether anti-inflammatory or regulatory cytokines are produced to control in response to the heightened inflammation or whether their production is directly elicited by the infection. In either case, it is clear that the production of anti-inflammatory and regulatory cytokines is insufficient to suppress or modulate the excessive proinflammatory response prior to death [35]. Regardless, obese mice infected with* F. tularensis* displayed a more intense cytokine storm in both plasma and local tissue causing increased* F. tularensis* susceptibility.

It is unsurprising that obese mice have elevated levels of adipokines compared with lean mice as the link between their production and obesity has been widely documented. However, only leptin was associated with the cytokine storm and reduced survival in our study, while resistin was only moderately affected and levels of adiponectin were reduced in infected obese animals, contrary to previous reports [20]. Indeed, in* in vitro *assay utilizing isolated macrophages, addition of leptin increased the production of proinflammatory cytokines following infection with* F. tularensis*. The specific signaling events leading to this increase remain to be determined; however, it is possible that there exists a synergy between the JAK/STAT signaling pathway activated by leptin and the inflammasome signaling pathway activated by* F. tularensis *infection.

Similar to previous reports, obese individuals had slightly higher levels of a number of proinflammatory cytokines though this did not reach statistical significance in our study [44]. However, they were significantly upregulated after a stimulus (infection) induced inflammation. The lack of statistical significance could be explained by a limitation in the sensitivity of our assay. Alternatively, despite the lack of a statistical significance, these slight changes may be recognized by a sensitive immune system and be capable of altering the biological response. Regardless, infection of both lean and obese mice activated the inflammatory response, as expected; however, in obese mice, the levels of many of these same inflammatory cytokines were significantly higher than in lean controls.

Interestingly, sCD40L and IL-21 were significantly downregulated in obese mouse serum prior to infection. sCD40L blocks monocyte activation and activates myeloid-derived suppressor cells (MDSCs) and regulatory T cells (Treg). MDSCs and Treg both function to suppress the immune response [45]. On the other hand, IL-21 is a pleotropic cytokine with both pro- and anti-inflammatory properties. We expect that, in this circumstance, the immunosuppressive functions of IL-21 to inhibit DC activation and maturation and/or induce the production of IL-10 from either T cells or B cells (B10 cells) are dominant [46]. The suppression of sCD40L and IL-21 would therefore serve to enhance inflammation in obese mice.

As stated, only female mice were challenged in this study due to our past experience in infectious disease and obesity studies and prior associative studies with sepsis disease [34, 39]. Our study associated the increased susceptibility to* F. tularensis *disease with the increased inflammation stimulated by leptin production. However, since male and female mice have different amounts of fat as well as adipokines [33–35], the susceptibility of male obese mice may differ from female obese mice. Indeed, we have observed differences in the susceptibility of lean males and females to* F. tularensis *independent of leptin (unpublished observation). Furthermore, since obese humans have an increased inflammatory response compared to lean individuals [34], we anticipate a similar response in obese tularemic patients; however, this remains to be determined.

Herein, we demonstrated that obese female mice are more sensitive to disease following* F. tularensis *infection. Indeed, obese mice had higher serum levels of inflammatory cytokines following* F. tularensis* compared to lean mice. In addition, obese female mice had higher levels of the inflammatory adipokine leptin and reduced levels of the anti-inflammatory adipokine adiponectin.* In vitro* studies demonstrated that exposure of macrophages to leptin resulted in increased inflammation in response to* F. tularensis *infection compared to macrophages infected with* F. tularensis *alone. This increased inflammation was observed by increased production of cytokine mRNA in infected tissues as well as increased serum levels of IL-6, IFN-*γ*, and TNF-*α*. These data lead to a model in which the expression of leptin from the activated hypertrophic adipocytes increases the activation of immune cells raising the inflammatory state of female obese mice similar to that reported for human obese females [44]. This higher than normal basal inflammation primes the immune system to generate an even more intense cytokine storm when elicited by* F. tularensis* infection.

## Data Availability

The data used to support the findings of this study are available from the corresponding author.

## Supplementary Materials

Supplementary Materials Supplemental Figure 1: Obesity-induced changes in plasma levels of inflammatory cytokines preinfection. Plasma levels of 20 cytokines were analyzed by multiplex ELISA and analyzed by ANOVA with Bonferroni posttest. Data is displayed as the mean and confidence intervals for the differences between the lean and obese animal groups (*∗*p<0.05 by two-way ANOVA). Supplemental Figure 2: IL-21 and sCD40L were reduced in the plasma of obese animals compared with lean mice. Prior to infection, only IL-21 and sCD40L were significantly altered in obese mice blood plasma compared with lean mice (*∗*p<0.05 by Mann–Whitney t-test). Supplemental Table: Detailed levels of plasma cytokines in lean and obese animals prior to (D0) and after infection with* F. tularensis*.

## References

[1] Henry T., Monack D. M. (2007). Activation of the inflammasome upon Francisella tularensis infection: Interplay of innate immune pathways and virulence factors. *Cellular Microbiology*.

[2] Mariathasan S., Weiss D. S., Dixit V. M., Monack D. M. (2005). Innate immunity against Francisella tularensis is dependent on the ASC/caspase-1 axis. *The Journal of Experimental Medicine*.

[3] Mares C. A., Ojeda S. S., Morris E. G., Li Q., Teale J. M. (2008). Initial delay in the immune response to Francisella tularensis is followed by hypercytokinemia characteristic of severe sepsis and correlating with upregulation and release of damage-associated molecular patterns. *Infection and Immunity*.

[4] Sharma J., Mares C. A., Li Q., Morris E. G., Teale J. M. (2011). Features of sepsis caused by pulmonary infection with Francisella tularensis Type A strain. *Microbial Pathogenesis*.

[5] Odegaard J. I., Chawla A. (2008). Mechanisms of macrophage activation in obesity-induced insulin resistance. *Nature Clinical Practice Endocrinology & Metabolism*.

[6] Weisberg S. P., McCann D., Desai M., Rosenbaum M., Leibel R. L., Ferrante A. W. (2003). Obesity is associated with macrophage accumulation in adipose tissue. *The Journal of Clinical Investigation*.

[7] Cinkajzlová A., Mráz M., Haluzík M. (2017). Lymphocytes and macrophages in adipose tissue in obesity: markers or makers of subclinical inflammation?. *Protoplasma*.

[8] Vandanmagsar B., Youm Y.-H., Ravussin A. (2011). The NLRP3 inflammasome instigates obesity-induced inflammation and insulin resistance. *Nature Medicine*.

[9] Koenen T. B., Stienstra R., Van Tits L. J. (2011). The inflammasome and caspase-1 activation: A new mechanism underlying increased inflammatory activity in human visceral adipose tissue. *Endocrinology*.

[10] Kretowski A., Ruperez F. J., Ciborowski M. (2016). Genomics and Metabolomics in Obesity and Type 2 Diabetes. *Journal of Diabetes Research*.

[11] Patsouris D., Li P.-P., Thapar D., Chapman J., Olefsky J. M., Neels J. G. (2008). Ablation of CD11c-positive cells normalizes insulin sensitivity in obese insulin resistant animals. *Cell Metabolism*.

[12] Sweeney G. (2002). Leptin signalling. *Cellular Signalling*.

[13] Cava A. L., Matarese G. (2004). The weight of leptin in immunity. *Nature Reviews Immunology*.

[14] Jernås M., Palming J., Sjöholm K. (2006). Separation of human adipocytes by size: hypertrophic fat cells display distinct gene expression. *The FASEB Journal*.

[15] Winkler G., Kiss S., Keszthelyi L. (2003). Expression of tumor necrosis factor (TNF)-*α* protein in the subcutaneous and visceral adipose tissue in correlation with adipocyte cell volume, serum TNF-*α*, soluble serum TNF-receptor-2 concentrations and C-peptide level. *European Journal of Endocrinology*.

[16] Acosta J. R., Douagi I., Andersson D. P. (2016). Increased fat cell size: a major phenotype of subcutaneous white adipose tissue in non-obese individuals with type 2 diabetes. *Diabetologia*.

[17] Aron-Wisnewsky J., Tordjman J., Poitou C. (2009). Human adipose tissue macrophages: M1 and M2 cell surface markers in subcutaneous and omental depots and after weight loss. *The Journal of Clinical Endocrinology & Metabolism*.

[18] Silswal N., Singh A. K., Aruna B., Mukhopadhyay S., Ghosh S., Ehtesham N. Z. (2005). Human resistin stimulates the pro-inflammatory cytokines TNF-*α* and IL-12 in macrophages by NF-*κ*B-dependent pathway. *Biochemical and Biophysical Research Communications*.

[19] Kuzmicki M., Telejko B., Szamatowicz J. (2009). High resistin and interleukin-6 levels are associated with gestational diabetes mellitus. *Gynecological Endocrinology*.

[20] Yang W-S., Lee W.-J., Funahashi T., Tanaka S., Matsuzawa Y., Chao C.-L. (2001). Weight reduction increases plasma levels of an adipose-derived anti-inflammatory protein, adiponectin. *The Journal of Clinical Endocrinology & Metabolism*.

[21] Osborn O., Olefsky J. M. (2012). The cellular and signaling networks linking the immune system and metabolism in disease. *Nature Medicine*.

[22] Hotamisligil G. S. (2006). Inflammation and metabolic disorders. *Nature*.

[23] Luchsinger J. A., Gustafson D. R., Bierhaus A. (2009). Adiposity, type 2 diabetes, and alzheimer's disease. *Journal of Alzheimer's Disease*.

[24] Dixit V. D. (2008). Adipose-immune interactions during obesity and caloric restriction: Reciprocal mechanisms regulating immunity and health span. *Journal of Leukocyte Biology*.

[25] Halberg N., Wernstedt-Asterholm I., Scherer P. E. (2008). The adipocyte as an endocrine cell. *Endocrinology and Metabolism Clinics of North America*.

[26] Dib L. H., Ortega M. T., Fleming S. D., Chapes S. K., Melgarejo T. (2014). Bone marrow leptin signaling mediates obesity-Associated adipose tissue inflammation in male mice. *Endocrinology*.

[27] Banks A. S., Davis S. M., Bates S. H., Myers M. G. (2000). Activation of downstream signals by the long form of the leptin receptor. *The Journal of Biological Chemistry*.

[28] Papathanassoglou E., El-Haschimi K., Li X. C., Matarese G., Strom T., Mantzoros C. (2006). Leptin receptor expression and signaling in lymphocytes: kinetics during lymphocyte activation, role in lymphocyte survival, and response to high fat diet in mice. *The Journal of Immunology*.

[29] Acedo S. C., Gambero S., Cunha F. G. P., Lorand-Metze I., Gambero A. (2013). Participation of leptin in the determination of the macrophage phenotype: An additional role in adipocyte and macrophage crosstalk. *In Vitro Cellular & Developmental Biology - Animal*.

[30] Lord G. M., Matarese G., Howard J. K., Baker R. J., Bloom S. R., Lechler R. I. (1998). Leptin modulates the T-cell immune response and reverses starvation- induced immunosuppression. *Nature*.

[31] Huttunen R., Syrjänen J. (2013). Obesity and the risk and outcome of infection. *International Journal of Obesity*.

[32] Atamna A., Elis A., Gilady E., Gitter-Azulay L., Bishara J. (2016). How obesity impacts outcomes of infectious diseases. *European Journal of Clinical Microbiology Infectious Diseases*.

[33] Gui Y., Silha J. V., Murphy L. J. (2004). Sexual dimorphism and regulation of resistin, adiponectin, and leptin expression in the mouse. *Obesity Research*.

[34] Vincent J.-L., Sakr Y., Sprung C. L. (2006). Sepsis in European intensive care units: results of the SOAP study. *Critical Care Medicine*.

[35] Sriskandan S., Altmann D. (2008). The immunology of sepsis. *The Journal of Pathology*.

[36] Zarkesh-Esfahani H., Pockley A. G., Wu Z., Hellewell P. G., Weetman A. P., Ross R. J. (2004). Leptin indirectly activates human neutrophils via induction of TNF-alpha. *The Journal of Immunology*.

[37] Karlsson E. A., Beck M. A. (2010). The burden of obesity on infectious disease. *Experimental Biology and Medicine*.

[38] Trivedi V., Bavishi C., Jean R. (2015). Impact of obesity on sepsis mortality: A systematic review. *Journal of Critical Care*.

[39] Jacobsson S., Larsson P., Johansson G. (2017). Leptin independently predicts development of sepsis and its outcome. *Journal of Inflammation*.

[40] Procaccini C., Jirillo E., Matarese G. (2012). Leptin as an immunomodulator. *Molecular Aspects of Medicine*.

[41] Iikuni N., Lam Q. L. K., Lu L., Matarese G., La Cava A. (2008). Leptin and inflammation. *Current Immunology Reviews*.

[42] Fantuzzi G., Faggioni R. (2000). Leptin in the regulation of immunity, inflammation, and hematopoiesis. *Journal of Leukocyte Biology*.

[43] La Cava A. (2017). Leptin in inflammation and autoimmunity. *Cytokine*.

[44] Maachi M., Piéroni L., Bruckert E. (2004). Systemic low-grade inflammation is related to both circulating and adipose tissue TNF*α*, leptin and IL-6 levels in obese women. *International Journal of Obesity*.

[45] Schlom J., Jochems C., Gulley J. L., Huang J. (2014). The role of soluble CD40L in immunosuppression. *OncoImmunology*.

[46] Croce M., Rigo V., Ferrini S. (2015). IL-21: a pleiotropic cytokine with potential applications in oncology. *Journal of Immunology Research*.

