# Supplementary material for: Obesity Exacerbates the Cytokine Storm Elicited by Francisella tularensis Infection of Females and Is Associated with Increased Mortality
Source: Biomed Res Int. 2018 Jun 26;2018:3412732. doi: 10.1155/2018/3412732 (PMC6038682; doi:10.1155/2018/3412732)
Supplement: Supplementary Materials — Supplemental Figure 1: Obesity-induced changes in plasma levels of inflammatory cytokines preinfection. Plasma levels of 20 cytokines were analyzed by multiplex ELISA and analyzed by ANOVA with Bonferroni posttest. Data is displayed as the mean and confidence intervals for the differences between the lean and obese animal groups (∗p<0.05 by two-way ANOVA). Supplemental Figure 2: IL-21 and sCD40L were reduced in the plasma of obese animals compared with lean mice. Prior to infection, only IL-21 and sCD40L were significantly altered in obese mice blood plasma compared with lean mice (∗p<0.05 by Mann–Whitney t-test). Supplemental Table: Detailed levels of plasma cytokines in lean and obese animals prior to (D0) and after infection with F. tularensis. [file 3412732.f1.pdf]

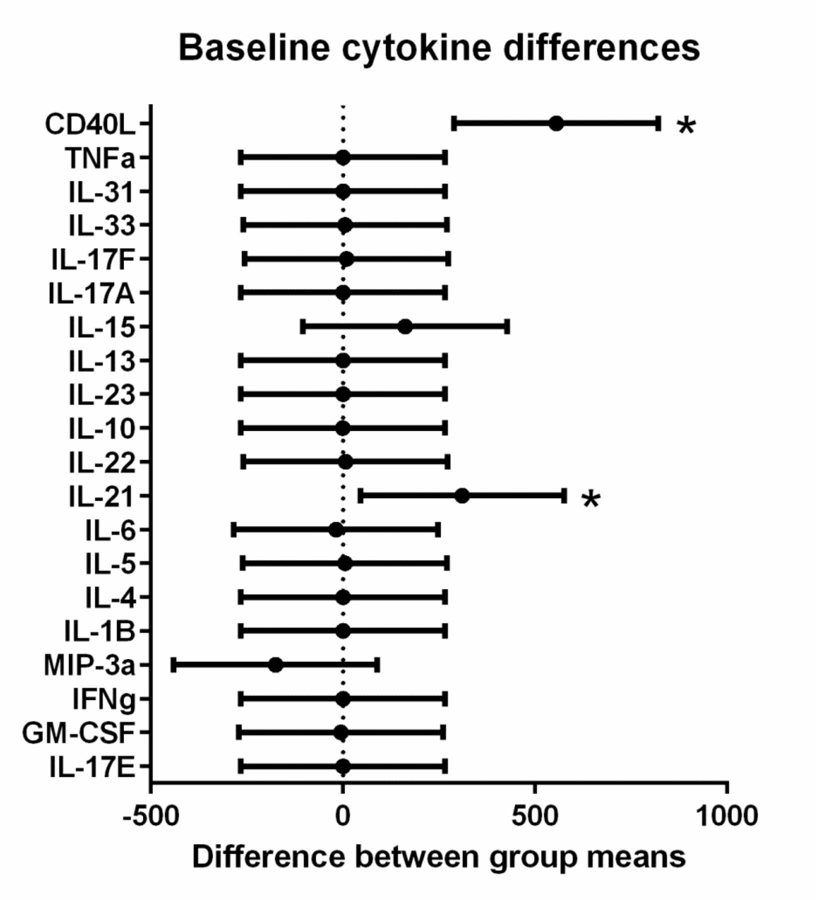

Supplemental Figure 1: Obesity-induced changes in plasma levels of inflammatory cytokines pre-infection. Plasma levels of 20 cytokines were analyzed by multiplex ELISA and analyzed by ANOVA with Bonferroni post-test. Data is displayed as the mean and confidence intervals for the differences between the lean and obese animal groups (*p<0.05 by two-way ANOVA).

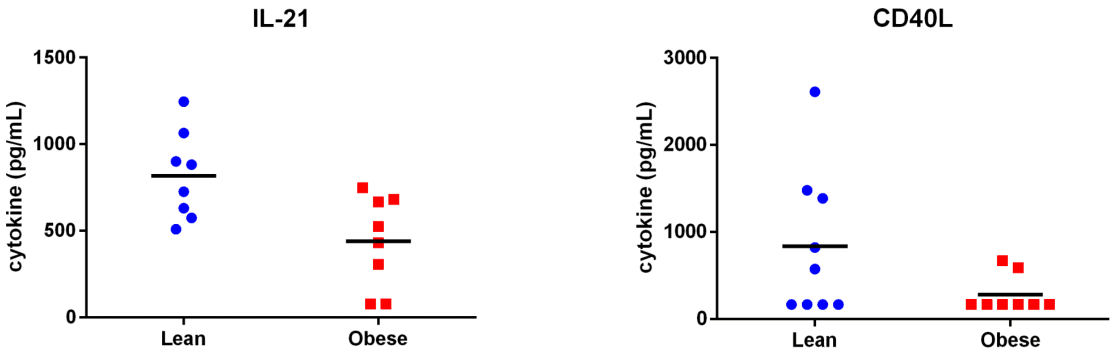

Supplemental Figure 2: IL-21 and CD40L were reduced in the plasma of obese animals compared with lean mice. Prior to infection, only IL-21 and CD40L were significantly altered in obese mice blood plasma compared with lean mice (*p<0.05 by Mann Whitney t test).

Supplemental Table: Detailed levels of plasma cytokines in lean and obese animals prior to (D0) and after infection with *F. tularensis*.

Table S1: Plasma cytokine concentrations

Cytokine	Mouse	D0	D3	D5	Terminus	*p-value
IL-1β	Lean	39.2 ± 0	78.0 ± 15.4	64.3 ± 5.3	137.8 ± 57.4	
	Obese	39.2 ± 0	80.8 ± 8.4	80.8 ± 15.0	162.2 ± 49.7	
IL-6	Lean	31.3 ± 0	**165.3 ± 69.2***	**152.1 ± 53.7***	1173.2 ± 637.4	<0.03
	Obese	49.1 ± 17.8	**1250.7 ± 600.8***	**730.9 ± 195.3***	1191.1 ± 546.1	
IFN-γ	Lean	31.3 ± 0	**672.5 ± 230.4***	**272.4 ± 98.4***	1748.9 ± 689.8	<0.02
	Obese	31.3 ± 0	**2375.8 ± 580.1***	**2376.3 ± 1063.7***	2676.4 ± 859.1	
TNF-α	Lean	8.4 ± 0	**21.7 ± 4***	**29.8 ± 7.7***	**68.1 ± 18.2***	<0.03
	Obese	48.8 ± 0	**83.5 ± 23.9***	**136.1 ± 56.0***	**239.5 ± 84.1***	
GM-CSF	Lean	137 ± 0	152.8 ± 12.5	159.6 ± 15.1	200.8 ± 28.7	
	Obese	142.1 ± 5.1	199.3 ± 36.1	263.5 ± 85.6	217.7 ± 47.5	
MIP-3α	Lean	195.0 ± 0	241.9 ± 17.5	200.4 ± 5.2	260.7 ± 47.6	
	Obese	270.7 ± 69.3	262.4 ± 26.2	253.4 ± 38.7	462.2 ± 133.4	
IL-21	Lean	**375 ± 51.7***	328.6 ± 33.7	394.5 ± 99.6	907.2 ± 358.7	<0.05
	Obese	**219.3 ± 46.8***	324.5 ± 21.1	352.5 ± 57.6	505.3 ± 107.2	
IL-15	Lean	181.1 ± 92.5	316.9 ± 45.0	485 ± 255.3	1395.4 ± 564.3	
	Obese	18.8 ± 0	425.9 ± 91.3	547.3 ± 192	626.3 ± 156.9	
IL-13	Lean	9.7 ± 0	282.0 ± 87	264.3 ± 65.8	406.2 ± 114.7	
	Obese	9.7 ± 0	396.5 ± 99.3	509.8 ± 126.7	460.9 ± 186.1	

Cytokine	Mouse	D0	D3	D5	Terminus	*p-value
IL-17A	Lean	156.0 ± 0	174.1 ± 11.5	170.4 ± 9.3	222.8 ± 28.5	
	Obese	156.0 ± 0	262.4 ± 45.6	271.2 ± 59.5	470.2 ± 221.7	
IL-17E	Lean	687.7 ± 0	9648.5 ± 1087.2	9014.1 ± 875.6	12181.5 ± 1513.2	
	Obese	687.7 ± 0	9901.7 ± 1131.5	12023.0 ± 2735.0	15359.1 ± 2901.1	
IL-17F	Lean	15 ± 9.2	69 ± 22.5	93.5 ± 42.5	225.4 ± 76.7	
	Obese	5.8 ± 0	104.5 ± 31.2	152.8 ± 59.0	188.2 ± 54.4	
IL-4	Lean	3.4 ± 0	6.7 ± 0.8	7.1 ± 1.1	14.5 ± 7.1	
	Obese	3.4 ± 0	6.9 ± 0.5	11.4 ± 5.1	18.7 ± 7.7	
IL-10	Lean	165.1 ± 0	106.7 ± 24.7	80.5 ± 2.5	136 ± 34.0	
	Obese	165.1 ± 0	137.5 ± 27.7	165.2 ± 45.8	289.2 ± 103	
IL-31	Lean	291.8 ± 0	775.8 ± 187.7	509.6 ± 178.3	681.0 ± 157.8	
	Obese	291.8 ± 0	755.7 ± 217.6	794.4 ± 311.8	932.6 ± 207.1	
IL-33	Lean	24.2 ± 5.7	380.6 ± 64.7	356.2 ± 43.2	992.2 ± 342.1	
	Obese	18.5 ± 0	395.6 ± 52.0	422.9 ± 76.3	540.3 ± 102.8	
IL-22	Lean	21.5 ± 15.8	39.1 ± 11.2	74.0 ± 28.7	288.1 ± 125	
	Obese	24.3 ± 9.9	96.5 ± 30.3	163.3 ± 85.1	542.4 ± 245.6	
IL-23	Lean	1367.0 ± 0	12704.9 ± 218.9	12486 ± 0	12502.4 ± 16.4	
	Obese	1367.0 ± 0	12842.3 ± 193.6	12574.7 ± 88.7	13874.1 ± 1285.8	
CD40L	Lean	**837.4 ± 282.1***	1024 ± 0	1024 ± 0	1024 ± 0	<0.05
	Obese	**282.3 ± 76.1***	1024 ± 0	1024 ± 0	1024 ± 0	

Table S2: Plasma adipocyte concentrations

Cytokine	Mouse	D0	D3	D5	Terminus	*p-value
Leptin	Lean	**309.9 ± 56.7***	**368.4 ± 67.9***	**607.7 ± 49.6***	**688.4 ± 230.8***	<0.05
	Obese	**809.3 ± 187.4***	**2259.5 ± 210.1***	**2326.8 ± 298.6***	**5949.3 ± 1138.3***	
Resistin	Lean	375.6 ± 59.7	1297.1 ± 170.9	908.7 ± 99.5	**1374.8 ± 225.8***	<0.02
	Obese	364.5 ± 79.8	896.3 ± 102.9	1103.2 ± 197.0	**2199.2 ± 164.0***	
Adiponectin	Lean	57535.4 ± 1419.4	**87773.2 ± 6596.5***	60152.0 ± 10022.9	70603.0 ± 10292.4	<0.002
	Obese	48758.5 ± 6475.5	**39803.6 ± 11430.8***	29415.4 ± 11974.9	82539.5 ± 7351.1	
